# Lyme in the prosthetic joint: two cases and a review of the literature

**DOI:** 10.5194/jbji-11-77-2026

**Published:** 2026-02-05

**Authors:** Hussam Tabaja, Matteo Passerini, Irene G. Sia, Elena Beam, Gina A. Suh

**Affiliations:** 1 Division of Public Health, Infectious Diseases and Occupational Medicine, Department of Medicine, Mayo Clinic, Rochester, MN, USA; 2 Department of Infectious Disease, Luigi Sacco Hospital, ASST-FBF-Sacco, Milan, Italy; 3 Department of Pathophysiology and Transplantation, University of Milano, Milan, Italy

## Abstract

Lyme periprosthetic joint infection (PJI) is rare, with only 8 cases reported in the literature. In this paper, we describe 2 cases and review the prior 8 cases to better characterize its diagnosis and management. All 10 cases presented with culture-negative PJI in Lyme-endemic regions and were confirmed to have Lyme PJI via a positive *Borrelia*-targeted polymerase chain reaction (PCR) run on joint specimens. Treatment strategies varied: 5 underwent debridement, antibiotics, and implant retention (DAIR); 1 had exchange arthroplasty; and 4 were managed non-operatively. Antimicrobial treatment also varied considerably. All patients achieved clinical cure. Lyme disease should be considered a potential cause of culture-negative PJI occurring in endemic regions. Because *Borrelia*-targeted PCR is not routinely included in standard PJI diagnostic workup, diagnosis may be delayed; therefore, clinicians should consider ordering this test when Lyme PJI is part of the differential.

## Key points

- Lyme PJI is rare but may be underdiagnosed in endemic areas.
- Diagnosis relies on synovial fluid or periprosthetic tissue Lyme PCR.
- Early diagnosis may allow medical therapy alone without surgical intervention.
- All reported Lyme PJI cases had favorable outcomes.

## 1 Introduction

*Borrelia burgdorferi*, the etiologic agent of Lyme disease, is the most common tick-borne pathogen in the United States (Nathavitharana and Mitty, 2015). Lyme disease progresses through distinct stages, including early localized (days to weeks from bite), early disseminated (weeks to months), and late disease (months to years) (Lantos et al., 2021). Arthritis is a well-recognized manifestation of late Lyme disease. It is a result of hematogenous seeding of the joint. Lyme arthritis is classically associated with native joints, while Lyme periprosthetic joint infection (PJI) has rarely been reported, with only 8 published cases to date. However, its prevalence may be underestimated, as *Borrelia* species are fastidious organisms, and Lyme PJI may represent a subset of culture-negative PJIs in endemic regions (Yang et al., 2025). Here, we present 2 cases of Lyme PJI and provide a summary of the previously reported literature. Notably, 1 of our cases represents the first reported instance of Lyme PJI involving a hip arthroplasty, as all previously published cases have involved the knee.

## 2 Methodology

Confirmation of infection in our cases was established using a qualitative *Borrelia*-targeted real-time polymerase chain reaction (PCR) (i.e., Lyme PCR) performed on synovial fluid or periprosthetic tissue (i.e., joint specimen). This laboratory-developed test was designed and validated by Mayo Clinic on synovial fluid and biopsy specimens from native joints in accordance with Clinical Laboratory Improvement Amendments (CLIA) standards. It has not been cleared or approved by the US Food and Drug Administration (FDA). The assay detects DNA from *B. burgdorferi*, *B. mayonii*, *B. afzelii*, or *B. garinii*.

Our literature search was conducted using PubMed, Embase, and Google Scholar, covering publications from 2005 to 2025. Search terms included “Lyme disease”, “*Borrelia* *burgdorferi*”, “prosthetic joint infection”, “periprosthetic joint infection”, and “arthroplasty infection”. We screened titles and abstracts, and full texts were reviewed when identified as relevant. We applied previously published classification criteria to categorize PJI onset as early (developing < 3 months from arthroplasty), delayed (3–12 months from arthroplasty), or late ( > 12 months from arthroplasty) and to further distinguish clinical presentations between acute (symptoms ≤ 3 weeks) and chronic (symptoms > 3 weeks) (Tabaja et al., 2024).

### 2.1 Case 1

A 76-year-old female, from Red Wing, Minnesota, was seen by orthopedic surgery (ORS) clinic in the summertime for left knee pain of 1 month's duration. She had a history of bilateral total knee arthroplasty (TKA) and bilateral total hip arthroplasty (THA). Her left primary TKA was done 10 years prior to presentation. Associated symptoms included knee warmth, swelling, and stiffness. However, she was still able to carry on her daily activities without limitation in range of motion. She denied systemic symptoms in the form of fevers, chills, and night sweats. Her serum C-reactive protein (CRP) was 26.7 mg L^−1^ (reference range ≤ 8.0), and her sedimentation rate (ESR) was 52 mm h^−1^ (reference range 0–29). X-ray of the knee showed a moderate knee joint effusion but a well-fixed implant with no radiographic loosening. A knee aspiration was positive for α -defensin. The sample amount was inadequate to run cell count analysis. The synovial fluid bacterial aerobic and anaerobic cultures remained negative.

She subsequently visited the infectious diseases (ID) clinic. The patient reported living in the countryside and enjoying regular strolls in a nearby wooded area. She did not recall any known injuries, skin pricks, or insect or tick bites. She also did not recall any preceding skin rash that might be consistent with erythema migrans (EM). She underwent a repeat left knee aspiration which showed 2012 white blood cells (WBCs), of which 57 % and 32 % were neutrophils and lymphocytes, respectively. Her synovial aspirate culture was sterile. Surprisingly, α -defensin testing on the repeat synovial sample was negative, and both serum CRP and ESR had normalized. Clinically, the patient also reported improvement in joint pain and swelling, which paralleled the normalization of her inflammatory markers. However, a synovial fluid Lyme PCR was positive for *B. burgdorferi*, confirming a diagnosis of Lyme PJI. Blood Lyme serologies were not checked, since the diagnosis was already made.

Due to minimal symptoms, the decision was to treat her conservatively with targeted antimicrobial therapy and without surgical intervention. She was treated with a 12-week course of oral doxycycline 100 mg twice daily. She completed the course and, on follow-up, her knee swelling, warmth, and pain completely resolved. Her CRP and ESR were also normal. She remained pain-free with preserved range of motion and knee function at 26 months of follow-up.

### 2.2 Case 2

A 67-year-old male, from Ontonagon, Michigan, was admitted to the hospital in the summertime for fevers, night sweats, and acute onset right hip pain of 2 weeks' duration. Associated symptoms included malaise, diffuse arthralgia, and myalgia. He underwent a right primary THA 5 months prior to presentation. He also had a history of mantle cell lymphoma for which he underwent autologous stem cell transplant in 2019, followed with maintenance rituximab until 2 months prior to his THA procedure. His serum CRP and ESR were 113 mg L^−1^ and 81 mm h^−1^, respectively. His peripheral total WBC count was normal. X-ray showed a well-fixed implant without radiographic evidence of loosening. A right hip arthrocentesis revealed 3549 WBCs with 88 % neutrophils and 10 % lymphocytes. Synovial α -defensin was negative. His synovial bacterial aerobic and anaerobic cultures remained negative. Also, a 16 s rRNA PCR (Broad Range Bacterial PCR (BRPCR)) performed on synovial fluid was negative. Because of his acute febrile presentation, he underwent debridement, antibiotics, and implant retention (DAIR) with modular component exchange. Intraoperatively, the synovial fluid was described as murky but without frank purulence. Five total intraoperative deep tissues were sent for bacterial aerobic and anaerobic cultures and for BRPCR, and all remained negative.

The patient was then seen by inpatient ID postoperatively. He reported recently spending time building a camp in a nearby wooded area. While he recalls having a wasp sting a few weeks prior to presentation, he denied any tick bites. He also denied any preceding skin rash. His outdoor exposure prompted adding a Lyme PCR to a periprosthetic tissue, and this turned positive for *B. burgdorferi*. A diagnosis of Lyme PJI was made. No blood Lyme serologies were tested. The patient was treated with 2 g of intravenous ceftriaxone daily for 2 weeks followed by 100 mg of doxycycline twice daily for 12 weeks. He had complete resolution of symptoms at the end of therapy, and both his CRP and ESR normalized. He was diagnosed with SARS-CoV-2 infection 1 month later and complained of concurrent right hip pain. His arthrocentesis had < 52 WBCs, ruling out a relapsed PJI. He had subsequent follow-up with resolution of right hip pain. He remained pain-free with a preserved range of motion and hip function at 23 months of follow-up.

## 3 Literature review

We identified 8 prior cases of Lyme PJI occurring in immunocompetent patients: 3 from Pennsylvania (Wright and Oliverio, 2016; Collins et al., 2017; Ali et al., 2021), 3 from Connecticut (Adrados et al., 2018; Miller et al., 2025), 1 from Wisconsin (Yang et al., 2025), and 1 from southwest Virginia (Saar and Fairbanks, 2024). Table 1 provides a summary of these cases.

**Table 1:** Clinical features of Lyme PJI.

Ref	Age/	Joint	Time from	Symptom	CRP	ESR	Blood serology	Blood serology	Synovial WBC	Synovial	Synovial	Joint	Surgery	Abx therapy	F/U	O/C
	gender		TJA (years)	duration (days)	(mg L^−1^)	(mm h^−1^)	(EIA)	(W Blot)	(cells µL )	PMN	α -defensin	*Borrelia* PCR		(weeks)	(months)	
1 (Yang et al., 2025)	70/F	TKA^a^	9	14^b^	27	54	NR	IgM ( + ) IgG ( + )	9478	83 %	NR	+	DAIR	DOX (4)	13	Res
2 (Wright and Oliverio, 2016)	67/M	TKA	1	90^c^	7	25	+	IgM ( - ) IgG ( + )	51 543	92 %	+	+	None	DOX (1) → CRO (6)	2	Res
3 (Collins et al., 2017)	83/M	TKA	6	3^b^	67	64	+	IgM ( - ) IgG ( + )	17 370	92 %	NR	+	TSE	DOX (2) → CRO (4)	12	Res
4 (Ali et al., 2021)	81/M	TKA^a^	15	2^b^	138	22	+	IgM ( + ) IgG ( + )	79 344	77 %	NR	+	DAIR	DOX (4)	6^d^	Res
5 (Adrados et al., 2018)	89/F	TKA^a^	16	1^b^	101	19	+	IgM ( + ) IgG ( - )	66 100	93 %	NR	+	DAIR	DOX (4)	5^e^	Res
6 (Adrados et al., 2018)	80/F	TKA^a^	0.3	NR	161	108	+	IgM ( - ) IgG ( - )	87 830	93 %	NR	+	DAIR	CRO (4) → DOX (4)	24	Res
7 (Miller et al., 2025)	68/F	TKA^a^	10	10^b^	36	33	NR	IgM ( - ) IgG ( + )	2319	71 %	NR	+	None	DOX (4)	24	Res
8 (Saar and Fairbanks, 2024)	68/M	TKA^a^	12	5^b^	61	8	NR	NR	14 760	90 %	NR	+	None	DOX (12)	5	Res
9 (Our case)	76/F	TKA^a^	10	30^c^	27	50	NR	NR	2012	57 %	+	+	None	DOX (12)	26	Res
10 (Our case)	67/M	THA^a^	0.4	14^b^	113	81	-	NR	3549	88 %	-	+	DAIR	CRO (2) → DOX (12)	33	Res

^a^ Had a well-fixed implant based on radiographic assessment or intraoperative inspection (no comment made about implant stability in remaining patients). ^b^ Acute PJI. ^c^ Chronic PJI.^d^ Knee symptoms resolved but patient died from ruptured brain aneurysm and stroke presumed to be due to neuroborreliosis. ^e^ Knee symptoms resolved but patient had cardiac-related death.Presenting symptoms ( n ): pain (10); swelling (10); fever (2); rash (0); wound dehiscence (0); wound drainage (0); sinus tract (0).Exposures ( n ): resides in endemic area (10); recalls tick bite/exposure (2).Abbreviations: Abx: antibiotics; CRO: ceftriaxone; CRP: c-reactive protein; DOX: doxycycline; EIA: enzyme-immunoassay; ESR: estimated sedimentation rate; F: female; F/U: follow-up; M: male; NR: not reported;O/C: outcome; PCR: polymerase chain reaction; Res: resolution of symptoms; THA: total hip arthroplasty; TJA: total joint arthroplasty; TKA: total knee arthroplasty; TSE: two-stage exchange;WBC: white blood cell; W Blot: western blot.

All had delayed or late monoarticular knee PJI. Seven patients presented with acute symptoms including knee pain and localized signs of inflammation, while one presented with chronic knee pain over 90 d (Wright and Oliverio, 2016). Six had a mild presentation in the absence of fever and were initially evaluated in the outpatient clinic (Yang et al., 2025; Wright and Oliverio, 2016; Adrados et al., 2018; Miller et al., 2025; Saar and Fairbanks, 2024). The remaining two patients had severe presentations; one presented with severe debilitating knee pain (Ali et al., 2021), and another presented with a febrile illness (Collins et al., 2017), necessitating direct admission and early surgical intervention. Neither patient had wound drainage, dehiscence, or a sinus tract. Furthermore, implant stability was reported in six patients, all of which had well-fixed prostheses. Only two patients recalled obvious tick exposure: one reported an “insect bite” on the neck 2 weeks prior to symptom onset (Yang et al., 2025), and the other, an avid hiker, noted frequent tick encounters over the preceding year (Miller et al., 2025). Furthermore, none of the patients reported EM at presentation or retrospectively.

All patients exhibited elevated serum and synovial inflammatory markers, resembling typical bacterial PJI. Lyme PJI was considered and worked up after conventional microbiology failed to identify a pathogen, ultimately confirmed by a positive Lyme PCR performed on a joint specimen. While *B. burgdorferi* was identified as the causative organism in seven cases, the species was not specified in one report (Saar and Fairbanks, 2024).

Four patients were managed with DAIR (Yang et al., 2025; Ali et al., 2021; Adrados et al., 2018), one was managed with two-stage exchange (TSE) (Collins et al., 2017), and three were treated non-operatively (Wright and Oliverio, 2016; Miller et al., 2025; Saar and Fairbanks, 2024). For the five operatively managed patients, the diagnosis of Lyme PJI was made after surgical intervention. In contrast, earlier recognition of Lyme disease and a mild clinical presentation may have persuaded clinicians to pursue antimicrobial therapy alone in the three medically managed cases.

Antimicrobial regimens varied, with treatment durations ranging from 4 to 12 weeks. The choice between oral doxycycline and intravenous ceftriaxone appeared arbitrary, and antibiotics were discontinued in all patients after a defined therapeutic course without transition to chronic suppressive therapy. All patients achieved favorable outcomes. Four patients had post-treatment inflammatory markers checked and had normalized (Yang et al., 2025; Wright and Oliverio, 2016; Ali et al., 2021; Adrados et al., 2018). In select cases, post-treatment joint aspiration was performed: synovial cell counts were assessed in two patients (Yang et al., 2025; Wright and Oliverio, 2016), and Lyme PCR was repeated in four (Yang et al., 2025; Wright and Oliverio, 2016; Collins et al., 2017; Ali et al., 2021). For all tested, synovial WBC counts normalized and repeat Lyme PCR was negative. No relapses occurred during follow-up.

## 4 Discussion

In this paper, we describe 10 cases of Lyme PJI, including 2 from our institution. Several key points about the clinical presentation, management, and outcomes of Lyme PJI could be discerned from our review.

All Lyme PJI cases presented with monoarticular involvement, with a clear predilection for the knee. One of our cases represents the first documented instance of Lyme PJI involving the hip. This pattern mirrors native Lyme arthritis, which most commonly affects the knee (Lantos et al., 2021). Most patients experienced delayed or late-onset acute PJI, and none had wound drainage, dehiscence, or sinus tract, which are features more typical of early postoperative or chronic PJI (Tabaja et al., 2024). When reported, implants were well fixed at presentation, which is another finding more typical of acute PJI (Tabaja et al., 2024). The severity of disease varied substantially across cases. Clinical severity varied, with some patients presenting mildly and managed initially as outpatients, whereas others required urgent surgical intervention.

A history of tick exposure or EM was rare, consistent with broader Lyme disease epidemiology. Only about 25 % of patients with Lyme disease recall a tick bite (Nadelman et al., 1996). Furthermore, although EM is a common finding (Steere and Sikand, 2003), it may be missing in a substantial minority of cases, and in the context of late Lyme arthritis, any preceding EM may have resolved well before joint symptoms emerged.

Inflammatory markers and synovial WBC counts resembled typical bacterial PJIs, often prompting empiric evaluation for common organisms first. *Borrelia* does not grow in routine culture, and Lyme PJI was typically considered only after cultures remained sterile.

Serologic Lyme testing alone is insufficient to distinguish remote from active infection. In native arthritis, a positive serologic test within the appropriate clinical context is generally considered sufficient to confirm the diagnosis (Lantos et al., 2021). However, in the uncommon scenario of Lyme PJI, we believe that a more definitive diagnosis is preferred and is established via direct detection of *B. burgdorferi* within the joint. PCR testing remains the most reliable method for confirming *Borrelia* within the joint (Nocton et al., 1994). Although the diagnostic performance of Lyme PCR on joint specimen has not been specifically evaluated in the context of PJI, it proved effective in all cases included in this review, confirming the diagnosis in each patient. Accordingly, Lyme PCR should be considered in culture-negative PJI in endemic areas, particularly when cultures are negative despite the absence of prior antibiotic exposure. However, PCR availability is limited, and clinicians may sometimes need to treat based on clinical suspicion and serology when testing cannot be performed.

Interestingly, BRPCR was negative in two cases where Lyme PCR was positive, though data remain too limited to determine comparative performance. These cases highlight the value of targeted Lyme PCR when suspicion persists despite negative broad-range testing. These cases also underscore the value of multidisciplinary evaluation in PJI, as involvement of ID specialists may increase consideration of atypical pathogens and facilitate targeted molecular testing when conventional workup is negative.

Management strategies varied. Some patients with mild presentations and early diagnosis were treated medically without surgery. Given the limited number of reported cases, this observation should not be interpreted as a general treatment recommendation. Surgical debridement continues to be the standard of care in PJI, and exceptions should involve shared decision-making and individualized risk assessment. Antimicrobial regimens resembled those used for native Lyme arthritis, though treatment duration and drug choice were inconsistent. No cases required chronic suppression.

All patients achieved clinical cure, with resolution of joint symptoms, regardless of the surgical or antimicrobial strategy employed. When obtained, post-treatment inflammatory markers normalized, and repeat aspirates demonstrated negative Lyme PCR and normalized cell counts. No relapses were observed during follow-up, which ranged from 2 to 33 months.

Traditionally, PJIs are considered incurable with antibiotics alone due to biofilm formation, necessitating surgical debridement or implant removal (Tabaja et al., 2024). However, the successful treatment of all four patients managed non-operatively raises the possibility that Lyme PJI may differ biologically from typical bacterial PJI; nevertheless, additional cases and further investigation into the biofilm-forming capacity of *Borrelia* species are needed to confirm this hypothesis.

## 5 Conclusion

In Lyme-endemic areas, clinicians should consider Lyme PCR in the diagnostic evaluation of culture-negative PJI, as direct detection of *B. burgdorferi* may guide therapy and prevent diagnostic delay. Multidisciplinary involvement, including ID consultation, may further support recognition of atypical pathogens and encourage early molecular testing. Lyme PJI appears to have favorable outcomes, and some mild cases have been successfully managed medically; however, this observation is based on a small number of reports and should not be interpreted as standard practice. Instead, it highlights an important area for future investigation.

## Data Availability

This study did not generate or analyze a new primary dataset. All data included in the literature review component were extracted from previously published case reports and case series that are publicly available in the peer-reviewed literature. The individual publications used as data sources are cited in the manuscript and listed in the reference section. No additional underlying datasets were created or deposited in public repositories as part of this study.
